# A Genome-Wide Integrative Association Study of DNA Methylation and Gene Expression Data and Later Life Cognitive Functioning in Monozygotic Twins

**DOI:** 10.3389/fnins.2020.00233

**Published:** 2020-04-09

**Authors:** Mette Soerensen, Dominika Marzena Hozakowska-Roszkowska, Marianne Nygaard, Martin J. Larsen, Veit Schwämmle, Kaare Christensen, Lene Christiansen, Qihua Tan

**Affiliations:** ^1^Epidemiology, Biostatistics and Biodemography, Department of Public Health, University of Southern Denmark, Odense, Denmark; ^2^Center for Individualized Medicine in Arterial Diseases, Department of Clinical Biochemistry and Pharmacology, Odense University Hospital, Odense, Denmark; ^3^Department of Clinical Genetics, Odense University Hospital, Odense, Denmark; ^4^Department of Biochemistry and Molecular Biology, University of Southern Denmark, Odense, Denmark; ^5^Human Genetics, Department of Clinical Research, University of Southern Denmark, Odense, Denmark; ^6^Department of Clinical Biochemistry and Pharmacology, Odense University Hospital, Odense, Denmark; ^7^Department of Clinical Immunology, Copenhagen University Hospital, Rigshospitalet, Denmark

**Keywords:** later life cognitive functioning, epigenome-wide association study, transcriptome-wide association study, integrative, monozygotic twins, intra-pair comparison

## Abstract

Monozygotic twins are genetically identical but rarely phenotypically identical. Epigenetic and transcriptional variation could influence this phenotypic discordance. Investigation of intra-pair differences in molecular markers and a given phenotype in monozygotic twins controls most of the genetic contribution, enabling studies of the molecular features of the phenotype. This study aimed to identify genes associated with cognition in later life using integrated enrichment analyses of the results of blood-derived intra-pair epigenome-wide and transcriptome-wide association analyses of cognition in 452 middle-aged and old-aged monozygotic twins (56–80 years). Integrated analyses were performed with an unsupervised approach using KeyPathwayMiner, and a supervised approach using the KEGG and Reactome databases. The supervised approach identified several enriched gene sets, including “neuroactive ligand receptor interaction” (*p*-value = 1.62^∗^10-2), “Neurotrophin signaling” (*p*-value = 2.52^∗^10-3), “Alzheimer’s disease” (*p*-value = 1.20^∗^10-2), and “long-term depression” (*p*-value = 1.62^∗^10-2). The unsupervised approach resulted in a 238 gene network, including the Alzheimer’s disease gene *APP* (Amyloid Beta Precursor Protein) as an exception node, and several novel candidate genes. The strength of the unsupervised method is that it can reveal previously uncharacterized sub-pathways and detect interplay between biological processes, which remain undetected by the current supervised methods. In conclusion, this study identified several previously reported cognition genes and pathways and, additionally, puts forward novel candidates for further verification and validation.

## Introduction

Cognitive functioning involves processes such as attention, action, learning, planning, memory, reasoning, problem-solving, communication, and decision making (Chan et al., 2008). In general, cognitive functioning of an individual matures and improves until early adulthood (Salthouse, 2010). Some measures of cognition, such as measures of effectiveness of processing performed at the time of assessment (e.g., speed and memory tests) starts to decline after early adulthood, whereas other measures of cognition, such as measures representing abilities acquired throughout life (e.g., vocabulary knowledge) increase at least until the sixth decade of life (Salthouse, 2010). At older ages, cognitive functioning in some individuals progresses from normal to mildly impaired, and sometimes further to dementia and neurodegenerative diseases like Alzheimer’s disease (AD) (Gan et al., 2018). Other individuals remain cognitively healthy, although the continued and inevitable decline in cognitive functioning with age potentially has a significant influence on their quality of life (Dause and Kirby, 2019).

The heritability of cognitive functioning has been reported to be considerable, in the range of 50–80% as estimated in twin studies (Pedersen et al., 1992; McGue and Christensen, 2002). Genome-wide association studies (GWAS) of general cognitive functioning have so far identified more than 300 associated genetic variants (e.g., Ibrahim-Verbaas et al., 2016; Sniekers et al., 2017; Davies et al., 2018; Savage et al., 2018; Hill et al., 2019a), however, despite this success, large parts of the heritability of cognitive functioning are still unaccounted for (Hill et al., 2019b). Other sources of biological variation that potentially contribute to the variation in a multi-factorial phenotype like cognition are variation in molecular markers of DNA methylation and gene expression (Trerotola et al., 2015). Analyses of genome-wide epigenetic and transcriptomic data in relation to cognitive functioning have mainly been performed in individuals with different degrees of cognitive impairment (i.e., elderly and/or diseased individuals) and not with a focus on cognitive functioning during normative cognitive aging in healthy middle-aged or young elderly individuals. However, recently Starnawska et al. (2017) published an epigenome-wide association study (EWAS) of 486 individuals from the survey of middle-aged and old-aged Danish twins also investigated in the present study: two CpG sites in the *ZBTB46* and *TAF12* genes were identified to have suggestive significant association (*p* < 1E-06) to cognitive function. Furthermore, enrichment analysis revealed the “Neuroactive ligand-receptor interaction” gene set to be the most enriched gene set (multiple testing corrected *p*-value = 0.0588). Additionally, in an EWAS meta-analysis of 11 cohorts from the CHARGE consortium, Marioni et al. (2018) investigated seven different measures of cognitive functioning and found two significant results: an association between cg21450381 located in an intergenic region on chromosome 12 and global cognitive functioning measured by the mini-mental state examination (*N* = 6,780), and an association between a CpG in the *INPP5A* gene and phonemic verbal fluency (*N* = 6,390). Marioni et al. (2018) did not perform enrichment analysis. Harries et al. (2012) performed a transcriptome-wide association study (TWAS) in 691 subjects from the InCHIANTI study (age range 30–104 years) and identified the *CCR2* gene as nominally significantly associated to cognitive function (multiple testing corrected *p*-value = 0.076). Enrichment analysis was conducted, but no significant gene sets were found. Finally, in a recent TWAS of 470 individuals from the survey of Danish middle-aged and old-aged twins also explored in the present study (Nygaard et al., 2019), the *POU6F1* gene was identified as associated with cognitive functioning with suggestive significance (multiple testing corrected *p*-value = 0.09). Gene set enrichment analysis conducted in this study identified gene sets related to ribosomal proteins, protein metabolism, RNA metabolism, the immune system, and infectious disease as significantly associated with cognitive function.

In addition to studies of one type of omics data at a time, an approach aiming to decipher the functional genomics of a given phenotype is the integration of different layers of biological variation, e.g., the integration of genome-wide genetic data (GWAS), genome-wide DNA methylation data (EWAS), and genome-wide gene expression data (TWAS) (Pineda et al., 2015). The most straightforward practice of integration of omics data involves the integration of two different kinds of data, e.g., genetic variants and gene expression data, or genetic variants and DNA methylation data for identifying expression and methylation quantitative trait loci (eQTLs and mQTLs), respectively (e.g., Yin et al., 2015; van Dongen et al., 2018). Integration of DNA methylation data and gene expression data has been commonly used to simply correlate the results obtained separately by EWAS and TWAS analyses (e.g., Gervin et al., 2012; Li et al., 2019). The rationale behind this approach is to exclude the potential complex interaction between DNA methylation and gene expression processes, while considering only genes of relevance to the phenotype of interest, i.e., restricting to sites with nominal significance below a chosen cut-off. The advantage of this approach is a lowering of the potential risk of false negative findings present when analyzing all sites in the dataset.

With the aim to investigate the functional genomics of cognitive functioning in later life, we integrate the results of an EWAS and a TWAS of intra-pair differences in cognition and intra-pair differences in blood-derived DNA methylation markers and in gene expression levels, respectively. The EWAS and TWAS are performed as intra-pair analyses in 452 healthy middle-aged and old-aged monozygotic twins resulting in a robust study design, where the genetic contribution to the phenotype and molecular markers can be controlled for. We apply a conventional supervised gene set enrichment analysis (GSEA) via the Kyoto Encyclopedia of Genes and Genomes (KEGG) and Reactome databases, as well as an unsupervised *de novo* pathway enrichment analysis using the algorithm KeyPathwayMiner. The advantage of the latter is that it can unravel sub-networks (also referred to as key pathways), which are aberrant with respect to, for instance, gene expression values compared to the organism’s interaction network (Alcaraz et al., 2014), and hence can identify previously uncharacterized sub-pathways (Alcaraz et al., 2016). Also, KeyPathwayMiner can detect interplay between biological processes, which remain undetected by the current GSEA methods that are based on a pre-defined list of pathways of known biological processes (Batra et al., 2017).

## Materials and Methods

### Study Population and Phenotype Data

The study population comprised 452 monozygotic twins drawn from the Study of Middle-Aged Danish Twins (MADT) conducted within the framework of the Danish Twin Registry. MADT was initiated in 1998 as a Danish nation-wide study of 4,314 twins randomly selected from the consecutive birth cohorts of 1931–1952. In 2008–2011 a follow-up study was performed on all eligible twin pairs originally enrolled (Pedersen et al., 2019). Zygosity was established by four questions regarding physical similarity, which correctly classified more than 95% of the pairs (Christiansen et al., 2003). The present study population included all monozygotic (MZ) twin pairs who participated in the MADT follow-up study and for whom genome-wide DNA methylation and gene expression data were available. Informed consent to participate in the survey was obtained from all participants and the survey was approved by The Regional Scientific Ethical Committees for Southern Denmark (S-VF-19980072) and conducted in accordance with the Helsinki II declaration.

General cognitive functioning was assessed by a cognitive composite score based on five brief cognitive tests related to verbal fluency (the number of animals named in a minute), immediate word recall (recall of items from a list of 12 words), delayed word recall (recall of items from the list of 12 words after approximately 10 min), forward digit recall (forward recall of series of numbers), and backwards digits recall (backwards recall of series of numbers) (McGue and Christensen, 2002). The verbal fluency and immediate and delayed word recall tests were adapted from the neuropsychological assessment proposed by the Consortium to Establish a Registry for Alzheimer’s Disease (CERAD) (Morris et al., 1989), while the forward and backwards digits recall tests reflect attention and working memory (McGue and Christensen, 2002). The scores from each of the five tests were standardized using the means and standard deviations (SDs) of the participants from MADT who were younger than 50 years of age at baseline in 1998, and the cognitive composite score was computed by summation of the five standardized scores. If one of the five items were missing, the cognitive composite score was multiplied by 5/4. When more than one item was missing the composite score was coded as missing. Finally, the composite score was linearly transferred to have a mean of 50 and a SD of 10.

### Biological Data

The genome-wide methylation data was extracted from a dataset of 492 MADT twins (Soerensen et al., 2019), while the genome-wide transcriptome data was extracted from a dataset on 496 MADT twins (Nygaard et al., 2019).

#### Genome-Wide DNA Methylation Data

DNA was isolated from buffy coat using the salt precipitation method and 500 ng DNA per sample was bisulfite converted using the EZ Methylation Gold kit (Zymo Research, Orange County, CA, United States). DNA methylation was measured using the Infinium HumanMethylation450K BeadChip (Illumina, San Diego, CA, United States). Quality control was conducted with the MethylAid (van Iterson et al., 2014) and Minfi (Aryee et al., 2014) R packages. Sample exclusion criteria: (a) <95% of probes with a detection *p*-value < 0.01, (b) failing the internal quality control probes of the MethylAid, or (c) failing verification of sex by multidimensional scaling of the X chromosome probe values. Four and two twin pairs were excluded due to poor sample quality and sex discrepancy, respectively. Probe exclusion criteria: (a) detection *p*-value > 0.01, (b) a raw intensity value of zero, (c) low bead count (<3 beads), (d) cross-reactive probes (Chen et al., 2013), or (e) measurement success rate < 95%. In total, 32,523 CpGs out of the 485,512 CpGs on the array were excluded, leaving 452,989 CpGs for analysis. Normalization was performed with Functional normalization (Fortin et al., 2014) using four principle components. To obtain a distribution more suitable for statistical testing (Du et al., 2010), *M* values were obtained by adding 0.001 to the methylation β values and logit transforming by the beta2m function (the lumi R package) (Du et al., 2008). Finally, a principle component analysis (PCA) of these data was performed (see Supplementary Figure 1 in the Supplementary Material 1) with the aim to investigate the correlation between potential technical and biological confounders and the principle components (PCs) and, subsequently, to include either the PCs or the confounding variable in the EWAS analysis (similarly to Tobi et al., 2015). The PCA showed that the top 4 PCs described the majority of the variance, as the subsequent PCs explained less than 2% of the variance (see Supplementary Figure 2 in the Supplementary Material 1). As PC1 correlated highly (correlation coefficient = 0.99) with sex, only PC2-4 were included in the statistical model (see section “Intra-pair Epigenome-Wide and Transcriptome-Wide Association Studies”). The DNA methylation data was annotated using GRCh37/hg19 using the annotation file and recommendations supplied by Illumina Inc. (Illumina, San Diego, CA, United States). For further details see Soerensen et al. (2019).

#### Genome-Wide Transcriptome Data

Whole blood was collected in PAXgene Blood RNA Tubes (PreAnalytiX GmbH, Hombrechtikon, Switzerland), total RNA was extracted by the PAXgene Blood miRNA kit (QIAGEN), and gene expression was examined by the Agilent SurePrint G3 Human GE 8 × 60K Microarray (Agilent Technologies). Sample labeling and array hybridization were performed by the ‘Two-Color Microarray-Based Gene Expression Analysis – Low Input Quick Amp Labeling’ protocol (Agilent Technologies); samples were labeled with Cy5 and the reference (a pool of 16 samples) was labeled with Cy3. The Agilent Feature Extraction software v. 10.7.3.1 (Agilent technologies) was used for array image analyses. The raw intensity data was background-corrected using the normexp method, within-array normalized by Loess normalization method, and between-array normalized by Quantile normalization (Yang et al., 2002; Yang and Thorne, 2003), all in the limma R package (Ritchie et al., 2007). The obtained data was applied to calculate log2-transformed Cy5/Cy3 ratios. Missing values were imputed by k-nearest neighbors averaging, and replicate probes were collapsed by calculation of the median. All the 50,599 probes on the array were annotated using the corresponding annotation file supplied by Agilent. For further details see Nygaard et al. (2019).

#### Cell Count Data

Blood leukocyte subtypes (monocytes, lymphocytes, basophils, neutrophils, and eosinophils) were counted for 443 of the 452 individuals using a Coulter LH 750 Haematology Analyser (Beckman Coulter, Woerden, Netherlands). For the remaining nine individuals, blood counts were imputed based on 450K DNA methylation data from 695 twins using a modified version of the PredictCellComposition method^1^ (see Soerensen et al., 2019 for details).

### Statistical Analyses

An intra-pair EWAS and an intra-pair TWAS of cognitive functioning were performed and the results of these analyses were subsequently used for the integrative analyses, first using a supervised GSEA employing the hypergeometric test for over-representation analysis of nominal significant findings in the KEGG and Reactome databases via the Molecular Signatures Database (MSigDB) database^2^ and second using an unsupervised *de novo* pathway enrichment analysis using the algorithm KeyPathwayMiner^3^ (Alcaraz et al., 2014).

#### Intra-Pair Epigenome-Wide and Transcriptome-Wide Association Studies

Both the EWAS and TWAS were performed by intra-pair analysis using a linear regression model (lm function) with the intra-pair difference in DNA methylation or gene expression level as the outcome variable, the intra-pair difference in the cognitive composite score as the explanatory variable, while adjusting for the sex, the mean age of the twin pair at time of blood sampling and the intra-pair difference in monocytes, lymphocytes, and eosinophils cell counts and, for the DNA methylation data, the intra-pair differences in PCs 2-4. Basophil counts were not included in the present statistical models due to low variability in the dataset, and neutrophil cell counts were not included as neutrophil and lymphocyte cell counts reflected much of the same variance (see Supplementary Figure 1 in the Supplementary Material 1). The intra-pair difference for a given covariate was calculated by subtracting the co-variate value of the twin with the lower cognitive composite score from the co-variate value of the co-twin with the higher cognitive composite score. The mean age of the twin pair at time of blood sampling was used, as the twins of a pair did not necessarily get their blood drawn on the same date. All analyses were carried out in R version 3.3.1 (scripts can be found in the Supplementary Material 1).

#### Pathway Analyses

For the pathway analyses the differentially methylated CpG sites and differentially expressed probes associated with cognitive function with p values below 0.05 and 0.01 in the EWAS and TWAS, respectively, were annotated to genes as described above. Consequently, with a *p*-value cut-off of 0.05, 20,732 CpG sites and 1,970 probes were found in the EWAS and TWAS, and these were annotated to 10,041 and 1,667 unique genes, respectively. The overlap between the EWAS and TWAS genes was 532 genes (the genes are listed in the Supplementary Table 1 in Supplementary Material 1), from hereon called the 532-gene overlap. Using a p value cut-off of 0.01 the corresponding numbers were 3,847 CpG sites and 333 probes, which were annotated to 2,776 and 279 unique genes, respectively, with an overlap of 25 genes (the genes are listed in the Supplementary Table 2 in Supplementary Material 1), from hereon called the 25-gene overlap.

##### Gene set enrichment analysis in MSigDB – the supervised method

GSEA was performed for the 532- and 25-gene overlaps, respectively, using the KEGG and Reactome databases (via MSigDB). Hypergeometric probabilities, or *p*-values, from the GSEA were corrected for multiple testing using the Benjamini–Hochberg false discovery rate (FDR) correction method (Benjamini and Hochberg, 1995), and only gene sets with an FDR < 0.05 are reported here.

##### *De novo* pathway enrichment analysis – the unsupervised method

KeyPathwayMiner is a *de novo* pathway enrichment method used to identify sub-networks (also referred to as key pathways), which are aberrant with respect to, for instance, gene expression values compared to the organism’s interaction network (Alcaraz et al., 2014). The output of this method is interaction networks of the submitted genes [in this case the 532- (*p*-value cut-off 0.05) and 25- (*p*-value cut-off 0.01) gene overlaps], as well as exception nodes, which are genes added by the algorithm to structure the network (i.e., they are not genes found to associate to the phenotype of interest in the initial analysis). The constructed networks can contain already known sub-pathways, yet also previously uncharacterized sub-pathways (Alcaraz et al., 2016). The following parameters were used for the KeyPathwayMinerWeb application^4^ : network: I2D (Brown and Jurisica, 2007), search strategy: INES (individual node exception), *L* = 0 and *K* = 2 or 4. L is the number of inactive samples, i.e., samples in which a given gene is not differentially expressed and methylated, and *K* is the number of inactive genes (also called exception nodes) allowed in a key pathway. Initially *K* values from 1 to 10 were tested (10 is the maximum value allowed in the KeyPathwayMiner). For the 532-gene overlap, key pathways with a *K* > 2 became highly complex and too dense for analysis, while for the 25-gene overlap the same was seen for *K* > 4. Consequently, *K* = 2 was chosen for the 532-gene overlap, and *K* = 4 was chosen for the 25-gene overlap. Cytoscape (Shannon et al., 2003) was used for visualization of the key pathways.

## Results

The characteristics of the study population can be found in Table 1. The EWAS and TWAS results for the individual CpG sites and probes can be found in Supplementary Tables 3a, 3b, 4 (Supplementary Material 2–4).

**TABLE 1:** Characteristics of the study population.

Number of individuals (number of twin pairs)	452 (226)
Female pairs (%)	101 (45)
Age, mean (*SD*)	66.27 (6.04)
Age range	56–80
Cognitive composite score, mean (*SD*)	45.60 (9.55)
Cognitive composite score, range	11.68; 84.93
Intra-pair cognitive difference, mean (*SD*)	6.89 (5.40)
Intra-pair cognitive difference, range	0.002–32.395

### Supervised Gene Set Enrichment Analysis

No gene sets displayed an FDR-corrected p value below 0.05 for the 25-gene overlap. Applying a less stringent cut-off of *p* < 0.1, the two KEGG gene sets “Neuroactive ligand receptor interaction” and “long-term depression” displayed nominal significance (*p*-value = 4.28E-04, FDR-corrected *p* value = 0.062, genes in overlap: *CRHR1*, *NMUR2*, *CHRNA10*, and *p*-value = 6.71E-04, FDR-corrected *p*-value = 0.062, genes in overlap: *CRHR1* and *PLCB1*, respectively). No Reactome gene sets were found using a cut-off of *p* < 0.1.

For the 532-gene overlap, the use of KEGG resulted in 26 enriched gene sets (FDR-corrected *p* –value < 0.05), including “Alzheimer’s disease,” “long-term depression,” “neurotrophin signaling pathway,” and “neuroactive ligand receptor interaction,” as well as gene sets related to metabolism, cellular processes, signal transduction, organismal systems (immune, endocrine, excretory, and sensory systems) and cancers, and cardiovascular diseases (see Table 2 and Supplementary Table 5 in Supplementary Material 1, for details). Similarly, GSEA of the 532-gene overlap using the Reactome database displayed 65 gene sets, which also related to metabolism, cellular processes, signaling pathways, the immune system, and disease, but also especially to hemostasis and transport of small molecules (see Supplementary Table 6 in Supplementary Material 1).

**TABLE 2:** Results of the gene set enrichment analysis of the 532-gene overlap with the supervised analysis using the KEGG database and FDR < 0.05.

**Hierarchy (BRITE)**		**Gene set name (KEGG)**	**K/k**	***p*-value**	***q* value**	**Genes in overlap**
Cellular Processes	Cell mobility	Regulation of actin and cytoskeleton (hsa04810)	216/14	2.79E-07	3.47E-05	PIK3R5, CRK, ITGA3, BDKRB2, INS, PIKFYVE, GIT1, GSN, TIAM2, BAIAP2, ITGAX, PFN1, RDX, ARHGEF7
	Transport and catabolism	Endocytosis (hsa04144)	183/9	3.07E-04	5.19E-03	HSPA2, ERBB3, PIKFYVE, GIT1, DNM3, GRK6, PSD4, ARAP3, HGS
	Cell growth and death	Apoptosis (hsa04210)	88/5	3.63E-03	2.94E-02	PIK3R5, CASP8, NGF, PRKAR1A, ATM
		Cell cycle (hsa04110)	128/6	3.87E-03	3.00E-02	ATM, TGFB1, SKP2, YWHAZ, MCM7, STAG1
Environmental Information Processing	Signal transduction	Calcium signaling (hsa04020)	178/12	1.30E-06	8.08E-05	NOS2, BDKRB2, PLCB1, ERBB3, NOS1, ATP2A2, ATP2A3, GNA11, ADRA1D, P2RX5, PDE1A, ITPKB
		MTOR signaling (hsa04150)	52/6	3.03E-05	1.13E-03	PIK3R5, INS, PDPK1, TSC2, EIF4E, HIF1A
		WNT signaling (hsa04310)	151/9	7.20E-05	2.03E-03	MAPK9, PLCB1, PPARD, DVL3, WNT3, PSEN1, CSNK2B, CTNNBIP1, CHD8
		MAPK signaling (hsa04010)	267/12	7.63E-05	2.03E-03	MAPK9, CRK, TGFB1, NGF, HSPA2, RASGRP2, CACNA2D1, DAXX, DUSP7, ELK4, MAPK8IP3, TAOK3
	Signaling molecules and interaction	Neuroactive ligand receptor interaction (hsa04080)	272/10	1.39E-03	1.62E-02	CRHR1, BDKRB2, ADRA1D, P2RX5, TSHR, CRHR2, MTNR1B, GABRQ, NMUR2, CHRNA10
Human Diseases	Cancers: Overview	Pathways in cancer (hsa05200)	328/17	3.73E-07	3.47E-05	PIK3R5, CRK, ITGA3, NOS2, MAPK9, HIF1A, PPARD, DVL3, WNT3, TGFB1, ETS1, CASP8, SKP2, RXRG, TPM3, NCOA4, DAPK3
	Cancers: Specific types	Renal cell carcinoma (hsa05211)	70/6	1.64E-04	3.05E-03	PIK3R5, CRK, PTPN11, TGFB1, HIF1A, ETS1
		Small cell lung cancer (hsa05222)	84/5	2.97E-03	2.91E-02	PIK3R5, NOS2, ITGA3, SKP2, RXRG
		Thyroid cancer (hsa05216)	29/3	4.51E-03	3.22E-02	TPM3, RXRG, NCOA4
	Cardiovascular diseases	Hypertrophic cardiomyopathy hcm (hsa05410)	85/5	3.13E-03	2.91E-02	ITGA3, ATP2A2, TGFB1, CACNA2D1, TPM3
		Dilated cardiomyopathy (hsa05414)	92/5	4.40E-03	3.22E-02	TGFB1, ITGA3, ATP2A2, CACNA2D1, TPM3
	Neurodegenerative diseases	Alzheimer’s disease (hsa05010)	169/8	8.41E-04	1.20E-02	PSEN1, PLCB1, NOS1, ATP2A2, ATP2A3, CASP8, PSENEN, SDHA
Metabolism	Lipid metabolism	Arachidonic acid metabolism (hsa00590)	58/5	5.64E-04	8.74E-03	GPX6, CYP2C9, CYP4F3, PTGDS, CBR1
	Amino acid metabolism	Arginine and proline metabolism (hsa00330)	54/4	3.55E-03	2.94E-02	NOS2, NOS1, ALDH2, P4HA3
	Carbohydrate metabolism	Inositol phosphate metabolism (hsa00562)	54/4	3.55E-03	2.94E-02	PLCB1, PIKFYVE, ITPKB, ISYNA1
Organismal Systems	Endocrine system	Insulin signaling (hsa04910)	137/10	4.90E-06	2.28E-04	PIK3R5, CRK, MAPK9, INS, PDPK1, TSC2, EIF4E, PRKAR1A, PYGB, PYGM
	Excretory system	Aldosterone regulated sodium reabsorption (hsa04960)	42/5	1.22E-04	2.52E-03	PIK3R5, PDPK1, INS, SCNN1B, ATP1A1
	Nervous system	Neutrophin signaling (hsa04722)	126/8	1.16E-04	2.52E-03	MAPK9, CRK, NGF, PSEN1, PIK3R5, PDPK1, PTPN11, YWHAZ
		Long-term depression (hsa04730)	70/5	1.33E-03	1.62E-02	PLCB1, NOS1, GNA11, CRHR1, PRKG1
	Immune system	Fc gamma r mediate phagocytosis (hsa04666)	97/6	9.50E-04	1.26E-02	PIKFYVE, DNM3, PIK3R5, CRK, GSN, DOCK2
		Chemokine signaling (hsa04062)	190/8	1.77E-03	1.94E-02	PLCB1, PIK3R5, CRK, DOCK2, GRK6, RASGRP2, TIAM2, GNB3
	Sensory system	Taste transduction (hsa04742)	52/4	3.10E-03	2.91E-02	GNB3, SCNN1B, PDE1A, TAS1R2

**q* value: FDR (false discovery rate) corrected *p*-value; K, number of genes in gene set; k, number of genes in overlap.*

### Unsupervised *de novo* Pathway Enrichment Analysis

**FIGURE 1:**
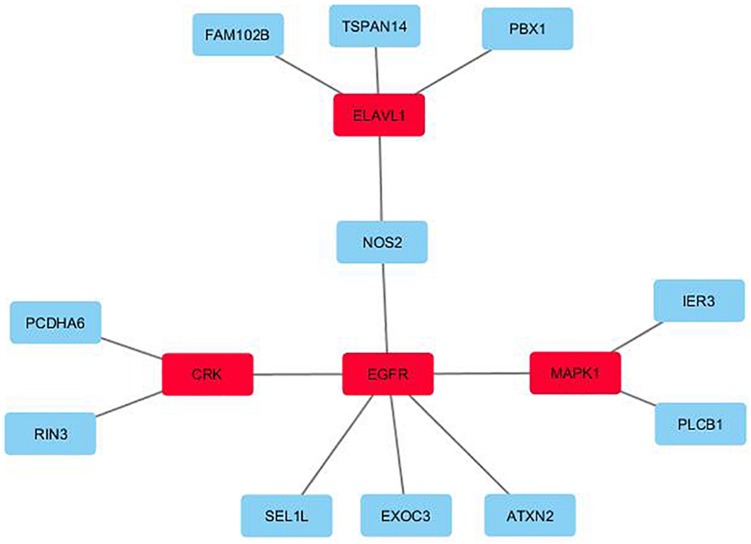
The key network identified by the unsupervised *de novo* pathway enrichment analysis for the 25-gene overlap. The exception nodes are highlighted in red, while the genes from the 25-gene overlap are highlighted in blue.

**FIGURE 2:**
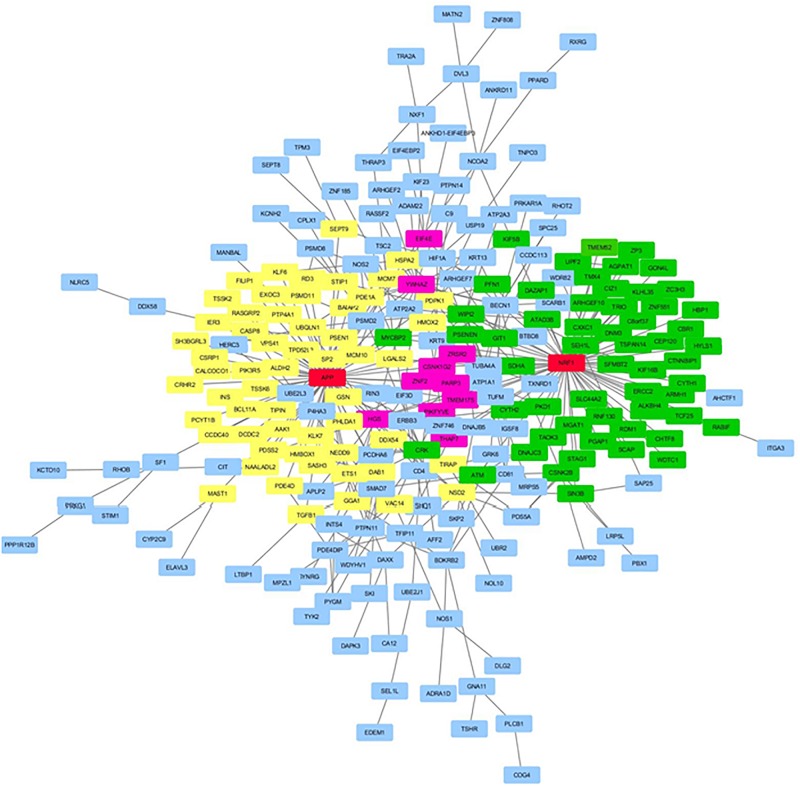
The key network identified by the unsupervised *de novo* pathway enrichment analysis for the 532-gene overlap. The exception nodes are highlighted in red, while the genes from the 532-gene overlap are highlighted in yellow (the genes directly connected to *APP*), green (the genes directly connected to *NRF1*), pink (genes directly connected to *APP* and *NRF1*), or blue (genes not directly connected to *APP* or *NRF1*).

Figure 1 displays the key network identified by the unsupervised (KeyPathwayMiner) method for the 25-gene overlap. The network contains 11 of the 25 genes of the 25-gene overlap, as well as four exception node genes, added by the algorithm to give structure to the network: the ELAV like RNA binding protein 1 gene (*ELAVL1*), the CRK proto-oncogene gene, adaptor protein gene (*CRK*), the epidermal growth factor receptor gene (*EGFR*), and the mitogen-activated protein kinase 1 gene (*MAPK1*). Correspondingly, the 532-gene overlap revealed a network containing 236 genes of the 532-gene overlap as well as two exception nodes: the Amyloid Beta Precursor Protein (*APP*) and the Nuclear Respiratory Factor 1 (*NRF1*) genes (see Figure 2). Of the 236 genes in the network, 57 genes were directly connected to *APP*, 57 genes were directly connected to *NRF1*, 10 genes were directly connected to both *APP* and *NRF1*, while 112 genes were not directly connected to *APP* nor *NRF1* (see Figure 2). All genes identified by the KeyPathwayMiner are listed in Supplementary Tables 7–9 in Supplementary Material 1.

## Discussion

In the present study we integrated the results of an EWAS and a TWAS of normative cognitive functioning in middle-aged and old-aged individuals with the aim to identify key pathways, which are distinct with respect to the biology behind cognitive function. To our knowledge, no study has previously applied such an approach.

In supervised gene set enrichment analyses (GSEA) of the 532 genes overlapping between the EWAS and TWAS (i.e., *p* < 0.05), the use of the KEGG database resulted in the identification of 26 gene sets (Table 2, FDR < 0.05), while the use of the Reactome database resulted in the identification of 65 gene sets (Supplementary Table 6, FDR < 0.05). Four KEGG gene sets related to neurological functioning, i.e., “neuroactive ligand receptor interaction” and “neurotrophin signaling,” or to diseases of the nervous system, i.e., “Alzheimer’s disease” and “long-term depression,” which appear to be of relevance for cognitive functioning (Table 2). The remaining 22 KEGG gene sets related to cellular processes, signal transduction, human diseases (cancer and cardiovascular diseases), metabolism (amino acid, carbohydrate, and lipid metabolism), and the endocrine, immune, excretory, and sensory systems. The 65 Reactome gene sets also related to signal transduction, metabolism, diseases (diabetes and HIV), immune function, and cellular processes (including the cell cycle, cell death, and transport), yet also to hemostasis, DNA replication, developmental biology, and extracellular matrix organization. Such a broad spectrum of biological functions identified in the present study indicates that the epigenetic and transcriptional control over cognitive functioning in late life involves multiple biological processes (as suggested by e.g., Konar et al., 2016).

The EWAS and TWAS published by Starnawska et al. (2017) and Nygaard et al. (2019), respectively, investigated study populations (*N* = 486 and *N* = 470, respectively) including the 452 twins in the present study population. Both studies performed intra-pair analysis and enrichment analyses of either EWAS or TWAS results, respectively, using KEGG gene sets in the former and Reactome gene sets in the latter. The enrichment analysis by Starnawska et al. (2017) reported four nominally significant gene sets related to neurological functioning, among these “neuroactive ligand receptor interaction,” which was also identified in the present study (Table 2). The TWAS by Nygaard et al. (2019) reported 81 significantly enriched Reactome gene sets for intra-pair difference in cognition, of which 15 were also observed in the present study (see Supplementary Table 6 in Supplementary Material 1). Interestingly, the ten gene sets related to the cell cycle identified in the present study were all reported by Nygaard et al. (2019). The remaining five gene sets found in both studies related to DNA replication, the immune system, signal transduction or diseases. Hence, the present study supports some of the gene sets previously found relevant for either variation in the methylome or in the transcriptome to be relevant for both levels of biological variation, but also puts forward novel gene sets.

The unsupervised method led to the identification of a network of 15 genes for the 25-gene overlap (Figure 1, *p*-value cut-off 0.01), and a network of 238 genes for the 532-gene overlap (Figure 2, *p*-value cut-off 0.05).

The genes of the 15-gene network first and foremost encode proteins taking part in processes relevant to cellular functioning and signal transduction^5^. All 15 genes are expressed in brain tissue and most have been reported as relevant for neurological functioning. The exception nodes added by KeyPathwayMiner, in order to give structure to the network, were the ELAV like RNA binding protein 1 (*ELAVL1*), the CRK proto-oncogene, adaptor protein (*CRK*), the epidermal growth factor receptor (*EGFR*), and the Mitogen-Activated Protein Kinase 1 (*MAPK1*). ELAVL1 is an mRNA stabilizing protein involved in, for instance, differentiation of embryonic stem cells, while CRK is an adapter protein binding tyrosine-phosphorylated proteins and involved in several signaling pathways, including cell migration. EGFR and MAPK1 are both protein kinases (EGFR a transmembrane and MAPK1 an intracellular kinase, respectively) involved in signaling cascades leading to, among others, cell proliferation. While *ELAVL1*, *EGFR*, and *MAPK1* are known from cancers, all four genes have been linked to neurological functioning: *CRK* is known from the Miller-Dieker Lissencephaly (‘smooth brain’) syndrome, characterized by abnormal brain structures, intellectual disability and seizures, while *MAPK1* has been linked to Alzheimer’s Disease (AD) (Lanke et al., 2018) and Retrograde Amnesia, which is defined as memory loss often following an injury or a disease. *EGFR* has been linked to dementia (Yokoyama et al., 2017), while mice with neuron-specific deletion of *ELAVL1* have been reported to have a phenotype resembling motor neuron disease (Sun et al., 2018). Of the 11 remaining genes (i.e., the genes originating from the 25-gene overlap) many have also been linked to neurological functioning, e.g., nitric oxide synthase 2 (*NOS2*), which synthesizes nitric oxide (NO), a free radical messenger believed to be involved in neurotransmission in the brain (reviewed in Džoljić et al., 2015). Protocadherin alpha 6 (*PCDHA6*) that belongs to the protocadherin alpha gene cluster encoding integral plasma membrane proteins, most likely plays a role in the synapsis formation and function in the brain (reviewed in Hamada and Yagi, 2001). Ataxin 2 (*ATXN2*) belongs to a group of genes associated with neurodegenerative diseases like amyotrophic lateral sclerosis, spinocerebellar ataxia-2, and Parkinson Disease (reviewed in Lee et al., 2018), while phospholipase C beta 1 (*PLCB1*) has been related to traits like epilepsy, depression, and AD (reviewed in Yang et al., 2016) and the Ras And Rab Interactor 3 (*RIN3*) has been linked to AD and dementia (Rasmussen et al., 2019). Animal models of *PBX1* (PBX Homeobox 1) have pointed to Parkinson’s disease (Villaescusa et al., 2016).

Nine of the genes in this 15-gene network were also present in the 238-gene network identified by KeyPathwayMiner when using the 532-gene overlap as input. The exception nodes added by KeyPathwayMiner for structure in this large network, the amyloid beta precursor protein (*APP*) and the nuclear respiratory factor 1 (*NRF1*), are both very relevant for cognitive functioning. *APP* encodes a transmembrane precursor protein, which is cleaved to form peptides, of which some form the basis of the amyloid plaques found in the brains of AD patients (reviewed in Tiwari et al., 2019). In addition, APP is known to play a role in cerebral amyloid angiopathy and is believed to be involved in processes related to neural cell adhesion, neuronal synaptic plasticity, synaptogenesis, and neurite growth (reviewed in Huang and Jiang, 2011). Also related to neurite growth, *NRF1* encodes a transcription factor activating the expression of proteins involved in cellular growth, development, respiration, heme biosynthesis, neurite outgrowth, and mitochondrial DNA transcription and replication, and is known to be involved in, among others, mitochondrial metabolism disease^6^. Its role in mitochondrial processes is considered to be important in relation to its role in neurodegenerative diseases (reviewed in Li et al., 2017), and it has been shown that the targets of NRF1 include genes involved in neurodegenerative diseases like Parkinson’s disease and AD (Satoh et al., 2013). In addition, the 238-gene network also contained presenilin 1 (*PSEN1*), a well-known interaction partner of *APP* (reviewed in Huang and Jiang, 2011).

*Post hoc* GSEA restricted to the 236 overlap genes present in the 238-gene KeyPathwayMiner network identified significantly enriched gene sets related to several different biological processes, including several signal transduction and cellular processes, but also cancer, metabolism, the immune system and hemostasis, as well as AD, neurotrophin signaling, gap junction, long-term depression, amyloid fiber formation, neutrophil degranulation, neurotransmitter receptors, and postsynaptic signal transmission, transmission across chemical synapses, and the neuronal system (see Supplementary Table 10 in Supplementary Material 1). No significantly enriched gene sets were identified based on the 11 overlap genes present in the 15-gene KeyPathwayMiner network, which, given the small size of the network, was to be expected. Finally, if focusing on the 57 and 57 overlap genes directly connected to either *APP* or *NRF1* in the 238-gene KeyPathwayMiner network, respectively, only one gene set, “axon guidance,” was found to be enriched for the *NRF1* connected genes, while 13 gene sets were found to be enriched for the *APP* connected genes; these were mainly related to signal transduction, cellular processes, cancer, metabolism, and “neurotrophin signaling” (see Supplementary Table 11 in Supplementary Material 1).

A potential limitation of the present study is the use of blood samples to study DNA methylation and gene expression of a phenotype primarily related to the brain. However, studies investigating the use of blood as a proxy for brain tissue when studying brain-related phenotypes have reported DNA methylation and gene expression status in the brain to mirror that in blood reasonably well (e.g., Aberg et al., 2013; Tylee et al., 2013). Nevertheless, future studies on the far less accessible brain tissue derived methylation and gene expression data and cognitive function are relevant to decipher the relevance of the pathways identified in the present study, specifically for the brain. Another limitation of the present study is the fact that probes/CpGs not annotated to genes are not included in the analyses, and hence the biological variation contributed by these sites is not considered. However, to include such CpGs in the present study would necessitate detailed information of the specific *cis*- or *trans*-action regulatory roles of each of such CpGs, which would be out of scope of the present study. Furthermore, as we did not want to introduce bias regarding annotation in the present study, we chose simply to annotate the CpGs and probes using the standard annotation files and recommendations by Illumina and Agilent, respectively. We do, however, realize that other annotation methods, for instance considering the trans regulatory roles of the CpGs, could give different results and consequently give rise to different sets of overlapping genes. One important strength of this study is the use of twin pairs; the intra-pair EWAS and TWAS enable us to explore genes associated with epigenetic or transcriptomic variation and cognition independent of genetic background and sheared early environment (Tan et al., 2015), significantly increasing the power of the study (Tan et al., 2017). Furthermore, as the correlation of cognitive functioning between the twins of a pair has been reported to decline with age, indicating that non-shared environmental and stochastic effects accumulate across lifespan potentially influencing the levels of cognitive functioning in aging individuals (McCartney et al., 1990), it supports the use of middle-aged and old-aged twins for studying the contribution of epigenetic and transcriptomic variation to the variation in cognitive functioning in later life. Finally, one might argue that the genes found to overlap between the EWAS and TWAS are simply chance findings. However, performing a *post hoc* permutation (shuffling the cognition values in the TWAS) did not identify the same genes as overlapping between the EWAS and the TWAS, and did not identify the same gene sets and networks (see Supplementary Tables 12–14 in Supplementary Material 1).

## Conclusion

This study identified biological pathways, which are distinct with respect to both epigenetic and transcriptional variations in relation to cognitive function in later life. Previously reported genes and pathways were confirmed, and novel candidates are put forward for further verification and validation. Hence, this study also promotes the use of unsupervised *de novo* network approaches for expanding our knowledge concerning the biology behind cognitive functioning in later life.

## Data Availability Statement

According to Danish and EU legislations, transfer and sharing of individual-level data require prior approval from the Danish Data Protection Agency. Our present local data protection rules do not allow individual-level data to be shared in public databases. For these reasons, the raw data cannot be deposited in a public database. However, we welcome any enquiries regarding collaboration and individual requests for data sharing. Requests can be directed to MS, msoerensen@health.sdu.dk.

## Ethics Statement

The study population comprised 452 monozygotic twins drawn from the Study of Middle-Aged Danish Twins conducted within the framework of the Danish Twin Registry. Informed consent to participate in the survey was obtained from all participants and the survey was reviewed and approved by The Regional Scientific Ethical Committees for Southern Denmark (S-VF-19980072) and conducted in accordance with the Helsinki II declaration.

## Author Contributions

MS and DH-R: conception and design of the study, performance of data and bioinformatics analyses, interpretation of results, project administration, and writing of the original draft. LC and MN: conception and design of the study, interpretation of results, project administration, and review and editing of draft. QT and LC: project administration, funding acquisition, interpretation of results, and review and editing of draft. VS: conception and design of the study. ML: data acquisition. KC: funding and data acquisition. All authors: read and approved the final version of the manuscript.

## Conflict of Interest

The authors declare that the research was conducted in the absence of any commercial or financial relationships that could be construed as a potential conflict of interest.

## Footnotes

1 https://github.com/mvaniterson/wbccPredictor

2 http://software.broadinstitute.org/gsea/msigdb

3 https://keypathwayminer.compbio.sdu.dk/keypathwayminer

4 https://keypathwayminer.compbio.sdu.dk/keypathwayminer

5 genecards.org

6 genecards.org
